# Supplementary material for: Ultrafast growth of nanocrystalline graphene films by quenching and grain-size-dependent strength and bandgap opening
Source: Nat Commun. 2019 Oct 24;10:4854. doi: 10.1038/s41467-019-12662-z (PMC6813332; doi:10.1038/s41467-019-12662-z)
Supplement: Supplementary file 1 — Supplementary Information [file 41467_2019_12662_MOESM1_ESM.pdf]

Supplementary Information for

Ultrafast growth of nanocrystalline graphene films by quenching and grain-size-dependent strength and bandgap opening

Zhao *et al.*

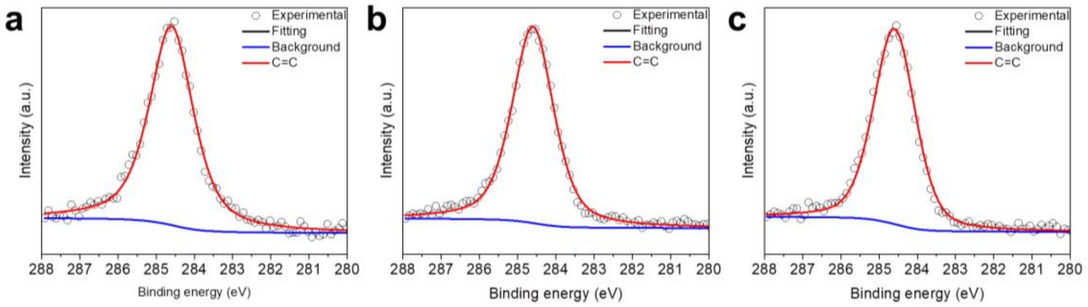

Supplementary Figure 1. XPS characterization of NG films and polycrystalline graphene films. XPS *C1s* spectra of as-synthesized 3.6-nm-grained NG (**a**), 10.3-nm-grained NG (**b**), and 500-μm-grained polycrystalline graphene film grown by conventional CVD on Pt foil with methane as carbon precursor (**c**). Note that all these samples show only a single *C1s* peak at 284.6 eV, corresponding to the graphite-like sp^2-hybridized carbon. These results confirm that the NG films are free of oxidation even though oxygen-containing ethanol is used as carbon source.

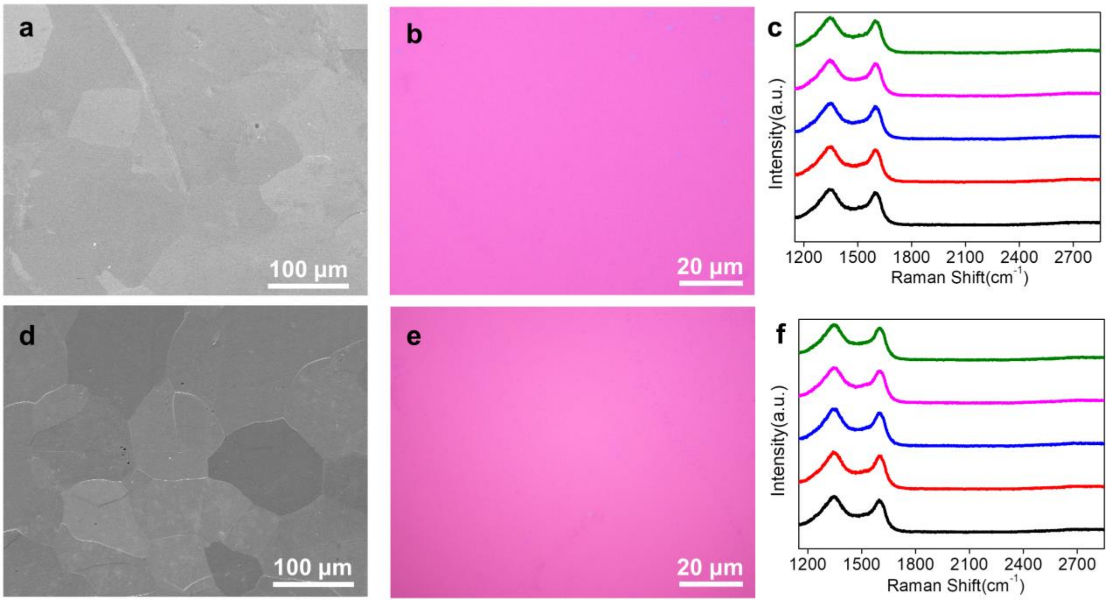

Supplementary Figure 2. Repeated use of Pt foil for NG film growth by ethanol quenching. a, SEM image of an as-grown NG film on Pt substrate. **b,** Optical image of the NG film transferred on SiO_2/Si. **c,** Raman spectra taken from randomly selected 5 positions in **b**. **d,** SEM image of the Pt substrate in **a** after transfer of NG. **e,** Optical image of the NG film grown on the repeated used Pt foil and transferred on SiO_2/Si. **f,** Raman spectra taken from randomly selected 5 positions in **e**.

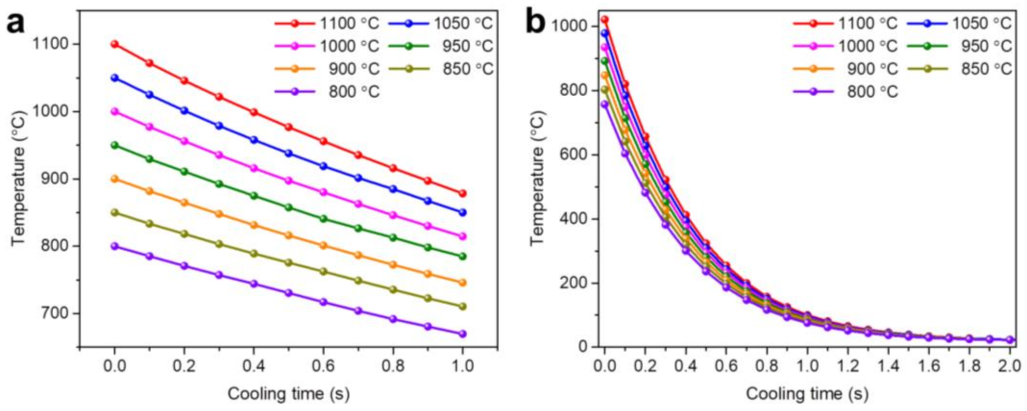

Supplementary Figure 3. Simulated cooling of Pt foils during the quenching process. The temperature profiles of 150-μm-thick Pt foils with different onset temperatures during cooling in air (**a**) and subsequently cooling in ethanol (**b**). Because it takes about 0.3 s to move the Pt foil from the heater coil into ethanol, we chose the period of 1 s to analyze the cooling process of Pt foil in air.

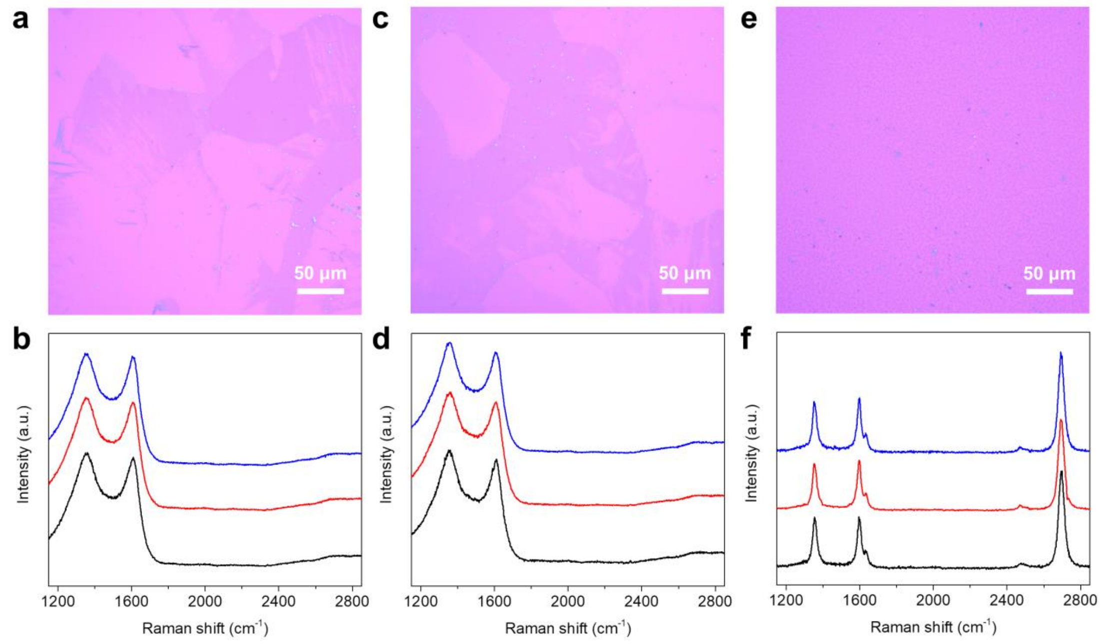

Supplementary Figure 4. Graphene grown with onset temperatures beyond 900 – 1050 ℃. Typical optical images and Raman spectra of the graphene films synthesized with different onset temperatures. **a, b,** 800 ℃. **c, d,** 850 ℃. **e, f,** 1100 ℃. The Pt foils used are 150 μm in thickness, and the samples have been transferred onto SiO_2/Si substrates by the bubbling method for characterizations.

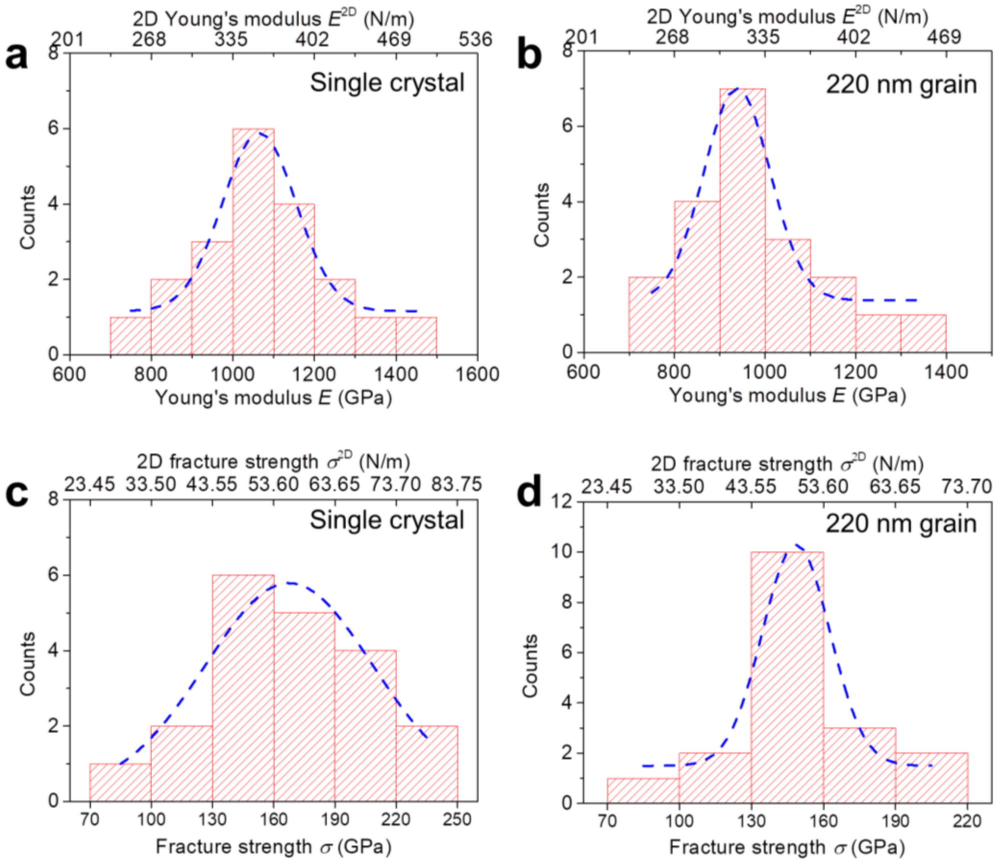

Supplementary Figure 5. Mechanical properties of single-crystal graphene and 220-nm-grained polycrystalline graphene. a,b, The histograms of the Young's modulus of single-crystal graphene (**a**) and polycrystalline graphene films with an average grain size of 220 nm (**b**). **c,d,** The histograms of the fracture strength of single-crystal graphene (**c**) and polycrystalline graphene films with an average grain size of 220 nm (**d**).

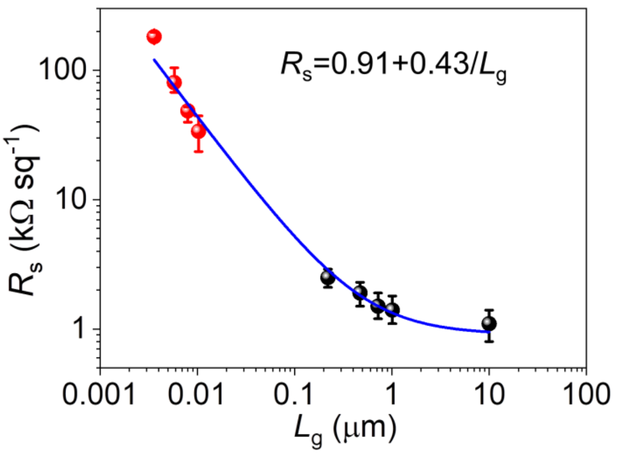

Supplementary Figure 6. The sheet resistance of graphene films as a function of grain size. The blue line is the fitting curve to the equation $R_s = R_s^G + \rho_{GB}/L_g$ for all data, and the fitted equation is shown in inset. The error bars show the variation of the sheet resistance.

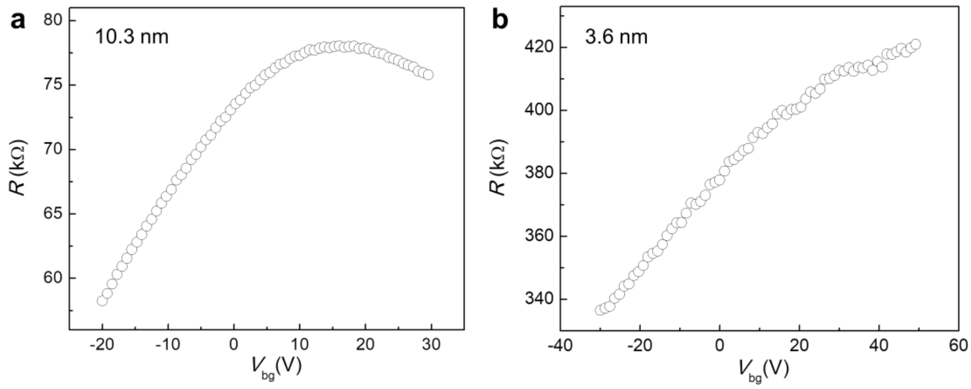

Supplementary Figure 7. Four terminal resistance as a function of back gate voltage of representative 10.3-nm grained (**a**) and 3.6-nm grained (**b**) NG films, measured at room temperature. The Dirac point of both NG samples is located in the positive gate voltage region, demonstrating hole doping of the graphene. The Dirac point shifts toward a more positive gate voltage with decreasing grain size because of the adsorption of more PMMA residues.

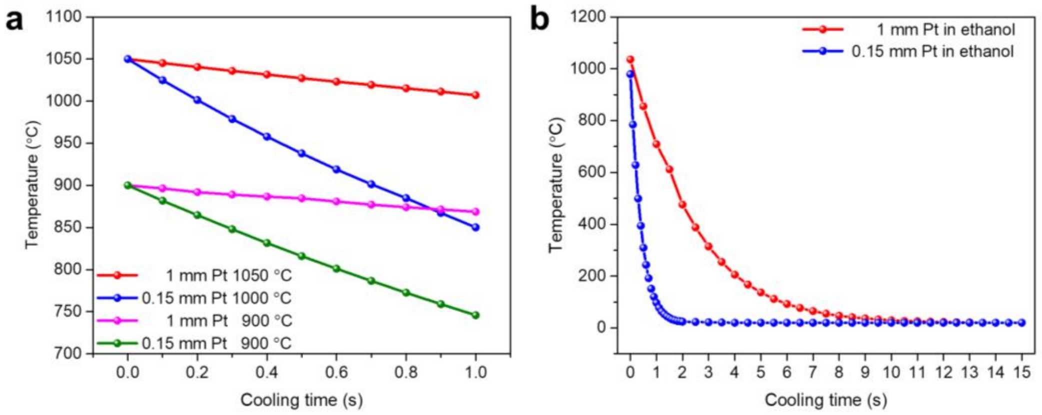

Supplementary Figure 8. The temperature profiles of Pt foils with different thicknesses and onset temperatures during cooling in air (**a**) and subsequent cooling in ethanol for onset temperature of 1050 ℃ (**b**).

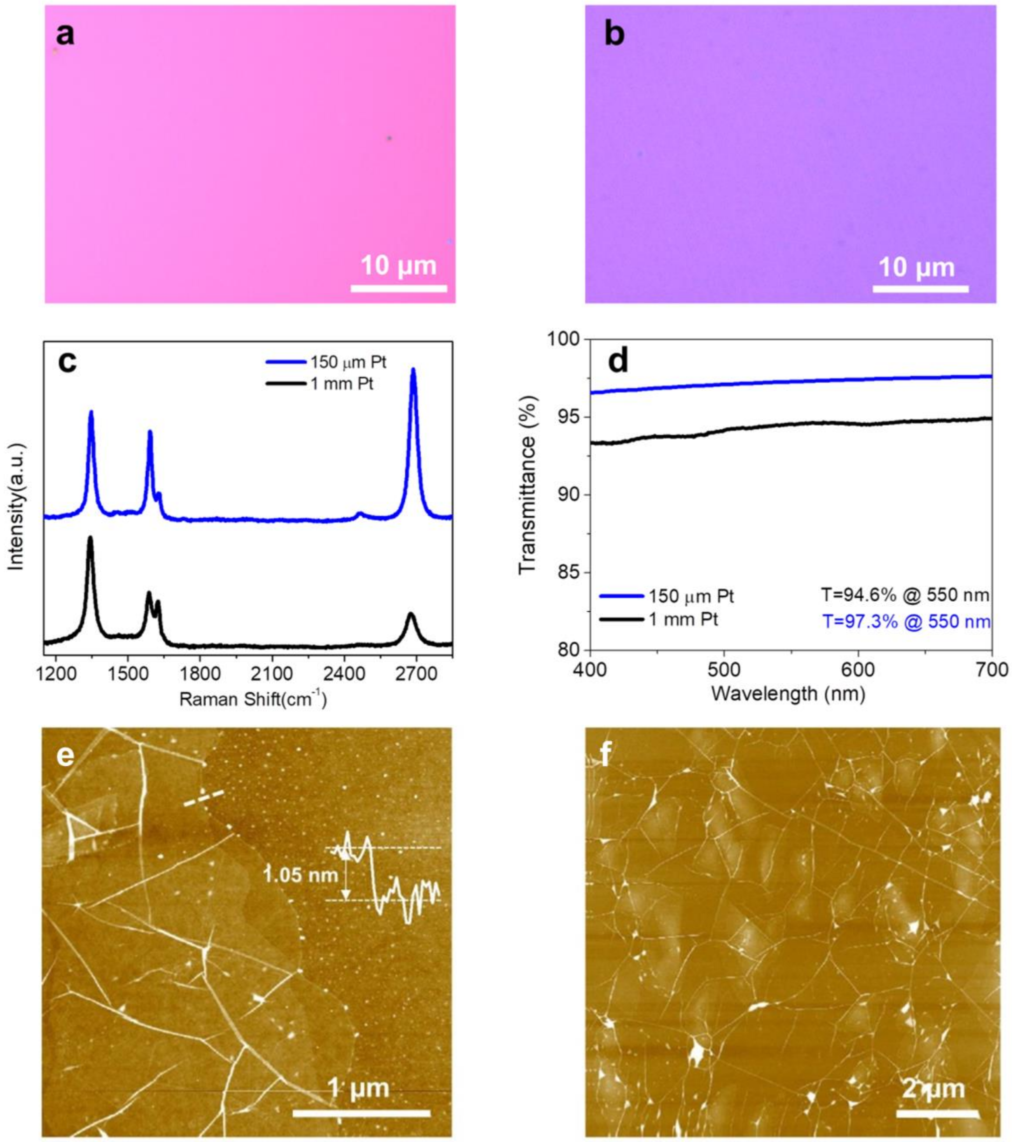

Supplementary Figure 9. The influence of the thickness of Pt foil on the structure of NG films. a,b, Optical images of the NG films grown on Pt foils with a thickness of 150 μm (**a**) and 1 mm (**b**) and transferred onto SiO_2/Si. **c,** Raman spectra of NG grown using Pt foils with a thickness of 150 μm (blue line) and 1 mm (black line). **d,** Optical transmittance spectra of the NG films grown using Pt foils with a thickness of 150 μm (blue line) and 1 mm (black line) measured by UV-vis-NIR spectrometer. **e,f,** AFM images of the NG films grown using Pt foil with a thickness of 1 mm.

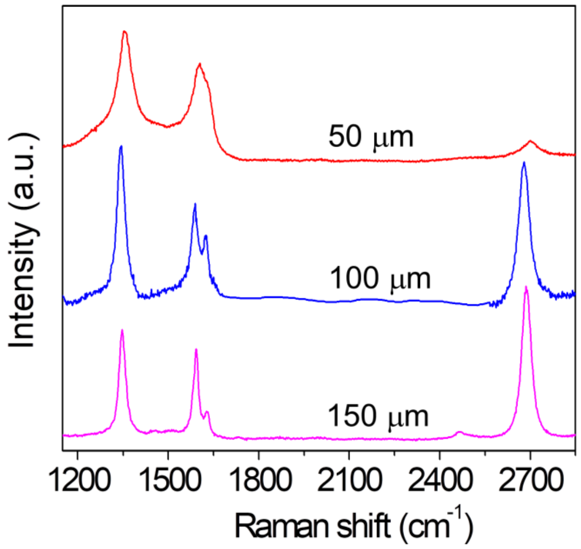

Supplementary Figure 10. The influence of the thickness of Pt foil on the synthesis of NG films. The onset temperature was 1050 ℃ for all three cases. Note that the NG films synthesized with a Pt foil of 100 μm and 50 μm thick show similar Raman spectra with those of 8.0-nm- and 5.8-nm-grained NG films (Fig. 3a,b in the main text), respectively.

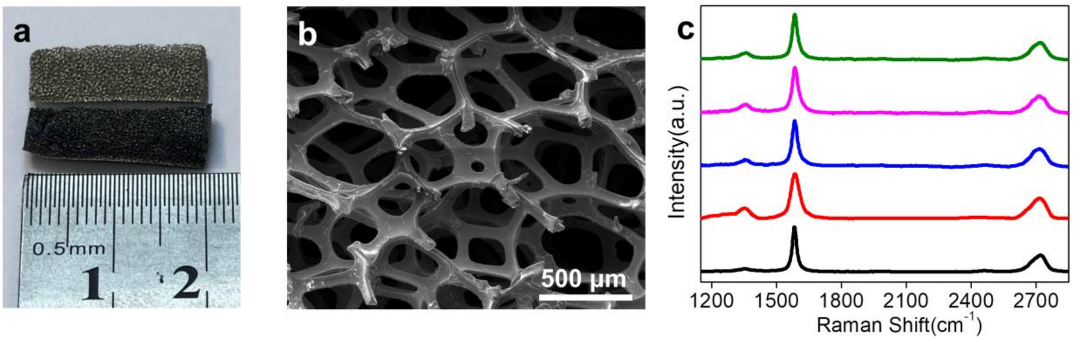

Supplementary Figure 11. Ultrafast growth of multilayer graphene foam on nickel foam by ethanol quenching. a, Photo of a pristine nickel foam (top) and a nickel foam grown with multilayer graphene by ethanol quenching (bottom), which show different colors because of the absence/presence of graphene multilayers. **b,** SEM image of the nickel foam grown with multilayer graphene. **c,** Raman spectra taken from randomly selected 5 positions in **b**, indicating the formation of high-quality multilayer graphene. The onset temperature of nickel foam is 1000 ℃.

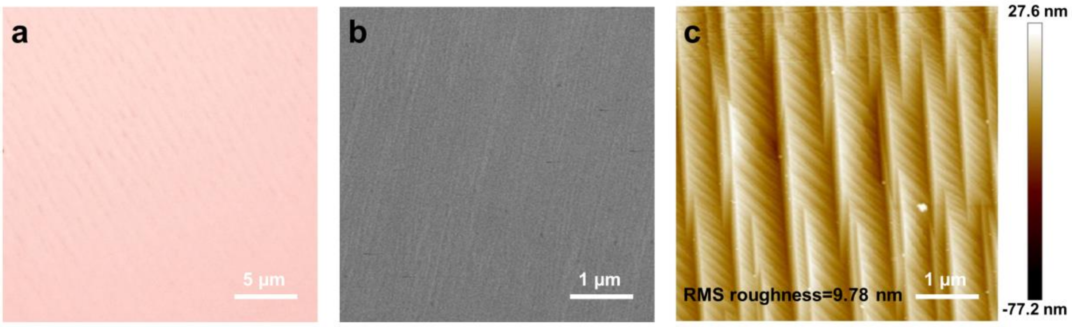

Supplementary Figure 12. Typical surface topography of Pt foil after polishing. **a,** Optical image. **b,** SEM image. **c,** AFM image.

Supplementary Table 1. The real onset temperature of 150-μm-thick and 1-mm-thick Pt foils with different onset temperatures during the ethanol quenching process, which are cooled in air for 0.3 s before immersing into ethanol

Thickness (mm)	Onset temperature (℃)	Real onset temperature (℃)
0.15	800	757
0.15	850	803
0.15	900	847
0.15	950	892
0.15	1000	935
0.15	1050	979
0.15	1100	1022
1.0	900	889
1.0	1050	1036
