# Supplementary material for: Ultrafast growth of nanocrystalline graphene films by quenching and grain-size-dependent strength and bandgap opening
Source: Nat Commun. 2019 Oct 24;10:4854. doi: 10.1038/s41467-019-12662-z (PMC6813332; doi:10.1038/s41467-019-12662-z)
Supplement: Supplementary file 3 — Description of Additional Supplementary Files [file 41467_2019_12662_MOESM3_ESM.pdf]

Description of Additional Supplementary Files

File Name: Supplementary Movie 1
Description: **Ultrafast growth of NG film on Pt foil by ethanol quenching.** The growth experiments were carried out in argon atmosphere. A piece of Pt foil was placed above an induction copper coil of an inductive heater (HFP-25, Rijia, China). The temperature of the Pt foil was monitored using an infrared thermometer and precisely controlled through a feedback control system. After heating to the set-up temperature, the Pt foil was rapidly quenched in ethanol at room temperature for a few seconds, and then taken out from ethanol for further characterizations.

File Name: Supplementary Movie 2
Description: **Ultrafast growth of multilayer graphene foam on nickel foam by ethanol quenching.** The growth experiments were carried out in argon atmosphere. A piece of nickel foam was placed above an induction copper coil of an inductive heater (HFP-25, Rijia, China). The temperature of the nickel foam was monitored using an infrared thermometer and precisely controlled through a feedback control system. After heating to the set-up temperature, the nickel foam was rapidly quenched in ethanol at room temperature for a few seconds, and then taken out from ethanol for further characterizations.
